# Ultrasounds assisted one-pot oxidative desulfurization of model fuel using green synthesized aluminum terephthalate [MIL-53(Al)]

**DOI:** 10.1038/s41598-023-40955-3

**Published:** 2023-08-22

**Authors:** Rodaina Metawea, Hassan A. Farag, El-Sayed El-Ashtoukhy, Mona M. Abd El-latif, Eman M. El-Sayed

**Affiliations:** 1 Alexandria Petroleum Company, Alexandria, Egypt; 2 Chemical Engineering Department, Faculty of Engineering, Alexandria University, Alexandria, Egypt; 3 Fabrication Technology Research Department, Advanced Technology and New Materials Research Institute (ATNMRI), City of Scientific Research and Technological Applications (SRTA-City), Alexandria, 21934 Egypt

**Keywords:** Energy science and technology, Materials science, Nanoscience and technology

## Abstract

Oxidative desulfurization (ODS) is considered to be one of the most promising desulfurization processes as it is energy-efficient and requires mild operating conditions. In this study, a novel green synthesized Al- based metal–organic framework with high surface area has been synthesized hydrothermally using waste polyethylene terephthalate bottles (PET) as a source of terephthalic acid as an organic linker. The prepared Al based MOF have been characterized using Fourier transform infrared spectroscopy (FTIR), X-ray diffraction (XRD), scanning electron microscope (SEM) and transmission electron microscopy (TEM). The catalytic activity of the prepared Al-MOF was evaluated in the oxidative desulfurization (ODS) of both modeled and real crude oil samples. The different operating parameters (temperature, time, catalyst dose, oxidant loading and sonication) on the ODS performance have been optimized. The optimal conditions for maximum removal of thiophene from modeled oil samples were found to be 30 min, 0.5 g of catalyst and 1:3 oil to oxidant ratio. Under the optimized conditions, sulfur removal in real oil samples obtained from Alexandria petroleum company was 90%. The results revealed that, the presented approach is credited to cost-effectiveness, environmental benignity, and ease of preparation, predicting great prospects for desulfurization of fuel oils on a commercial level.

## Introduction

The global crude oil consumption as a main source of energy is increasing continuously as a result of the fast economic development and population growth, which directed researchers to develop new desulfurization systems to improve crude oil quality and reduce its sulfur content^1^. High sulfur content has detrimental effects in the refining process, as it cause erosion of the pipelines and the refining equipment, also its dangerous influence on the environment. One of the common industrial desulfurization approaches in petroleum refineries is hydro-desulfurization (HDS), nevertheless HDS technique is expensive, needs high temperatures and pressures, and in addition it is not suitable for removing aromatic heterocyclic sulfur complexes such as thiophenes^2^. Solvent extraction, adsorption, oxidation^3^, ionic liquid are alternatives to hydro-desulfurization method^4–6^. Adsorption is the a promising technique to reduce sulfur content using various adsorbing materials which are able to remove the sulfur organic compounds depending on physical and chemical properties. Effective adsorbents materials should have high surface area, and proper pore size distribution^7^. Wide variety of adsorbents have been used for this purpose such as activated carbon^8^, zeolites^9,10^. Oxidative desulfurization (ODS) is a very promising method to deeply remove larger organosulfur compounds from oil and has received increasing attention in recent years^11^. Generally, the ODS process is operated under mild conditions, catalyzed by suitable catalysts. The sulfur-containing compounds are oxidized by selected oxidants to form corresponding sulfoxides or sulfones, which have higher polarity and are easy to be extracted from oil^12^. Several key features of Metal–organic Frameworks enable their use as efficient heterogeneous catalysts, in particular the exceptionally large surface area and porosity, the good thermal stability and the adjustable acidity/basicity^13–15^. Furthermore, some additional properties of these materials play a fundamental role in promoting ODS^16,17^. On the other hand, the polyethylene terephthalate (PET) materials are mass consumed for living and caused serious environmental problems. The environment issues of waste PET bring out with large volume after disposed and take long time in degradation. The recovery of terephthalic acid from used PET can be complete in different ease and economical means.

Ultrasound-assisted oxidative desulfurization is an innovative green approach for rapid, economical and safe removal of sulfur compounds under mild conditions. Generally, ultrasonic radiation is considered to be an attractive and efficient way used to accelerate the chemical reactions^18^. When the reaction medium is radiated with high-intensity ultrasounds, shock waves create acoustic cavitation in the medium. The development and consequent powerful bubbles collapse create spots with high temperature (up to 5000 K) and pressure (up to 1000 atm) in reaction medium^19^. The oxidative desulfurization accomplished in the biphasic system including extracting solvent and oil that ultrasound radiation can be scatter emulsion-like of biphasic system. The main objective of our work is to define a system for the desulfurization of fuels by using catalytic oxidative desulfurization using a green prepared Al based-MOF as catalyst. Al-based MOFs, or Metal–Organic Frameworks, are often preferred over other metal-based MOFs for several reasons. Firstly, aluminum is abundant and cost-effective, making it more accessible for large-scale production. Additionally, Al-based MOFs exhibit excellent stability and robustness, ensuring their durability and suitability for various applications. Furthermore, aluminum has a low toxicity profile compared to certain other metals, making Al-based MOFs safer for use in various industries and environmental settings. Overall, the advantages of Al-based MOFs in terms of availability, stability, and safety make them a favorable choice over other metal-based MOFs.In our green methodology, the used polyethylene terephthalate (PET) bottles have successfully been used as starting material in place of terephthalic acid for preparing PET-Al based MOF^20^. The prepared MOF was used a catalyst in a catalytic desulfurization process. The effect of different parameters (temperature, time, catalyst dose, oil to oxidant ratio and ultrasound waves) on % reduction of sulfur from modeled oil were studied. The optimized conditions will be applied for sulfur removal from real oil samples obtained from Alexandria Petroleum Company.

## Materials and methods

Aluminum nitrate nonhydrate (98%), terephthalic acid (≥ 98%) both from India, were all obtained from Loba chemie and were used as received. Acetonitrile ultra-gradient grade from France, hydrogen peroxide (30%) from Egypt, ethanol (99.8%) from UK, n-Hexane from UK and Thiophene (AR) from India. Real crude oil sample was obtained from Alexandria Petroleum Company with the specifications given in Table 1.

**Table 1:** The main specifications of the investigated real crude oil.

Density (g/ml)	0.8688
Pour point (°C)	− 6
Initial sulfur content % (m)	2.26%
Carbon content	5.68

### Aluminum terephthalate [MIL-53(Al)] synthesis

PET-MIL-53(Al) was synthesized by solvothermal technique using PET bottles as an organic linker. In a typical synthesis, used PET bottles were collected, washed, and cut into small flakes. About 2 g of PET flakes, 4 g of Al(NO_3_)_3_·9H_2_O, and 48 mL of DI water were ultrasonically mixed for 30 min. Then, the mixture was transferred to a teflon autoclave and heated at 215 °C for 8 h. After cooling to room temperature, the product was collected washed with hot ethanol and water respectively; finally, the resulting powder was dried at 110 °C for 24 h. The obtained powder was calcinated to 400 °C for 4 h to obtain white PET-MIL-53(Al) powder.

### MOF characterization

X-ray powder diffraction (XRD, Cu-K radiation, Shimadzu-7000, Kyoto, Japan) was used to determine the crystallographic phase of the prepared samples. Their morphology was examined by scanning electron microscopy (SU-70, Hitachi, Tokyo, Japan) combined with energy dispersive X-ray analysis (EDX) for elements identifications. The microstructure was also observed by JEM-2100 transmission electron microscope (TEM, JEOL, Japan) Fourier transform infrared (FTIR) analysis was done with a Bruker ALFA spectrometer (Bruker Corporation, Ettlingen, Germany). X-ray photoelectron spectroscopy (XPS) data were recorded on aESCALAB 250 instrument (Thermo fisher Scientific, USA). The specific surface area of the prepared MOFs can be determined using the Brunauer, Emmett, and Teller (BET) method. This method involves the physical adsorption of nitrogen gas onto the solid surface and quantifying the amount of adsorbate gas that corresponds to a monomolecular layer on the surface. The adsorption is driven by relatively weak interactions (van der Waals forces) between the gas molecules and the adsorbent surface of the samples. Surface area Brunauer, Emmett and Teller (BET) the physical adsorption of an N_2_ gas on the surface of the solid and the amount of adsorbate gas corresponding to a monomolecular layer on the surface are used to calculate the specifc surface area of the prepared MOF. A volumetric or continuous fow approach (Quantachrome Corporation Nova 1000, version 6.11 high speed, gas sorption analyzer) at 77 K can be used to determine the amount of gas adsorbed. Before the measurements, the samples were activated at 150 °C for 6 h.

### Oxidative desulfurization process (ODS)

The ODS reaction was carried out using a two-phase extraction-oxidation procedure. Thiophene was completely dissolved in n-hexane to make model oil with sulfur concentration of 500 ppm. In a typical reaction, 0.5 gm of catalyst was dispersed in 50 ml of acetonitrile in a 500 mL three neck bottom round bottom flask. To the dispersion, 50 ml model oil was added. The mixture was stirred for 10 min using magnetic stirrer then appropriate amount of H_2_O_2_ was added to start the oxidation reaction. After the start of the reaction, the stirring was stopped at consistent intervals, the samples from the upper oil phase were analyzed and the sulphur concentrations in the samples were determined by GC–MS. (ARL OPTIM’X WDXRF spectrometer) according to ASTM D2622. The % removal of the sulfur was calculated according to Eq. (1):

1 $$R=\frac{{\mathrm{C}}_{o}-{\mathrm{C}}_{f}}{{\mathrm{C}}_{o}}*100$$

where, R is the desulfurization rate, C_o_ is the initial sulfur concentration of the oil, and C_f_ is the final sulfur content of the oil after desulfurization.

## Results and discussion

### Aluminum terephthalate [PET-MIL-53(Al)] characterization

**Figure 1:**
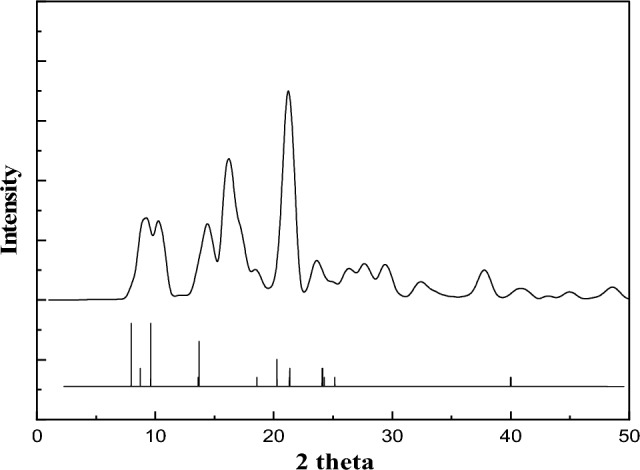
XRD patterns of the prepared PET-MIL-53(Al).

As presented in Fig. 1, the XRD patterns exhibit distinct peaks at specific 2θ angles typically 7°, 10°, 13° and 20° consistenting with the simulated ones (CCDC No. 181153)^21,22^ confirming the effective preparation of PET-MIL-53(Al).

**Figure 2:**
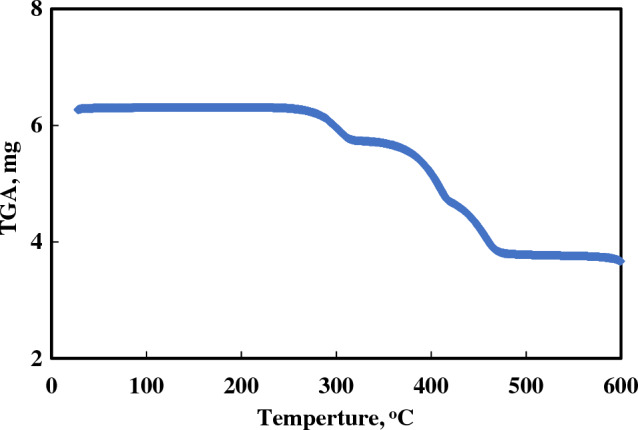
TGA curve of the prepared PET-MIL-53(Al).

The thermal property of the prepared PET-MIL-53(Al) was analyzed by TG measurements. According to Fig. 2, the weight loss of PET–MIL-53(Al) began around 260 °C, proving its high thermal stability during organic catalytic processes. There was only about 0.6 wt% weight loss at temperatures lower than 200 °C as seen in the figure, which is attributed to the removal of water and small molecules.

**Figure 3:**
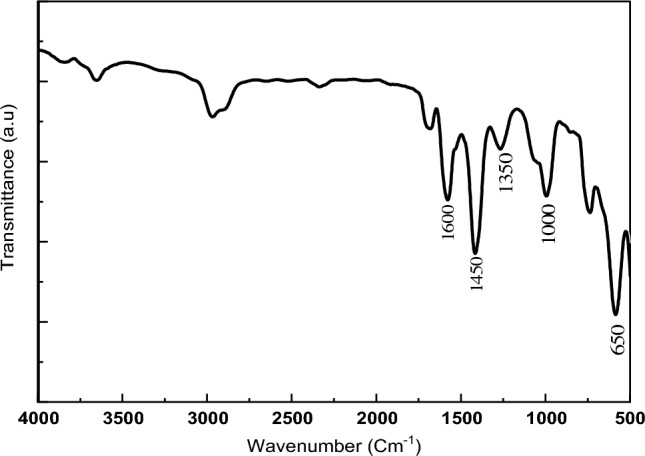
FTIR spectra of as-synthesized PET-MIL-53 (Al).

The Fourier transform infrared spectra of the PET-MIL-53(Al) are given in (Fig. 3). The presence of strong band around 1600 cm^−1^ corresponds to the stretching vibrations of carboxylate groups in the coordinated terephthalate molecules. Furthermore, a distinct IR band at 1450 cm^−1^ is attributed to C–C stretching modes. Additionally, the in-plane C–H bending is observed at 1000 cm^−1^, while the out-of-plane C–H bending occurs at 650 cm^−1^^21,23^.

**Figure 4:**
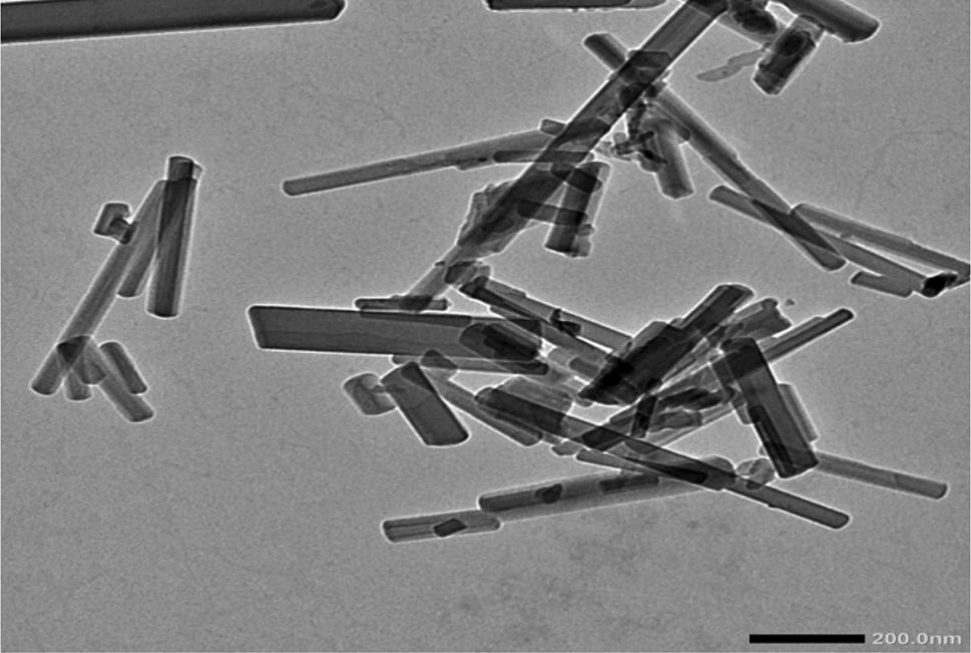
TEM images of PET-MIL-53 (Al) nanorods.

**Figure 5:**
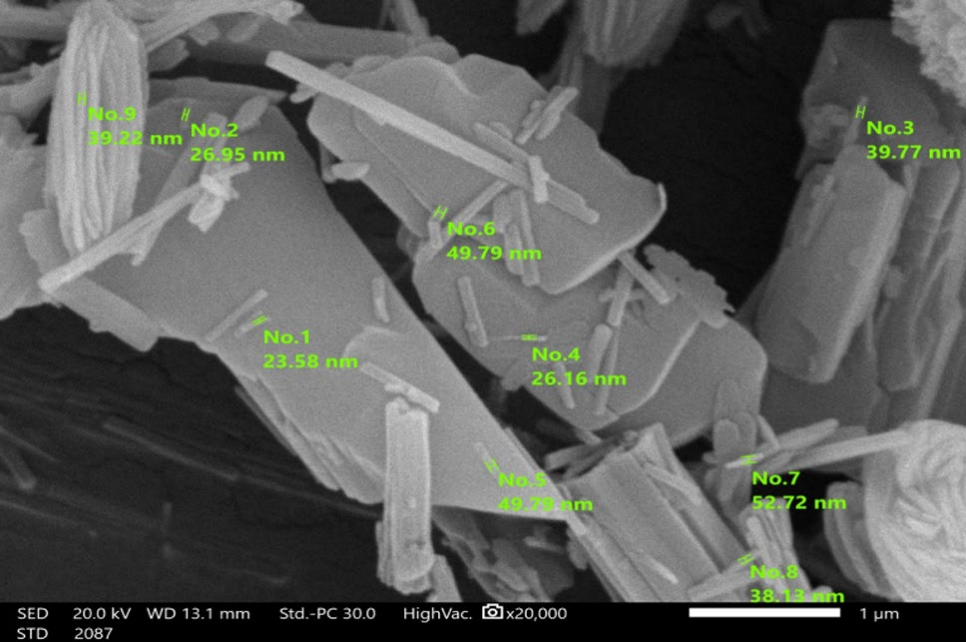
SEM images of PET-MIL-53 (Al) nanorods.

The TEM and SEM images (Figs. 4 and 5) were used to to define morphometric characteristics of the prepared PET-MIL-53 (Al) nanostructures. The TEM images showed elongated rod-like structures with a high aspect ratio, indicating the formation of nanorods. The images provided a visual representation of the nanorods' shape and confirmed their elongated morphology. The SEM images also revealed the surface morphology of the prepared material is nanorods. They showed elongated rod-like structures with varying lengths and diameters.

**Figure 6:**
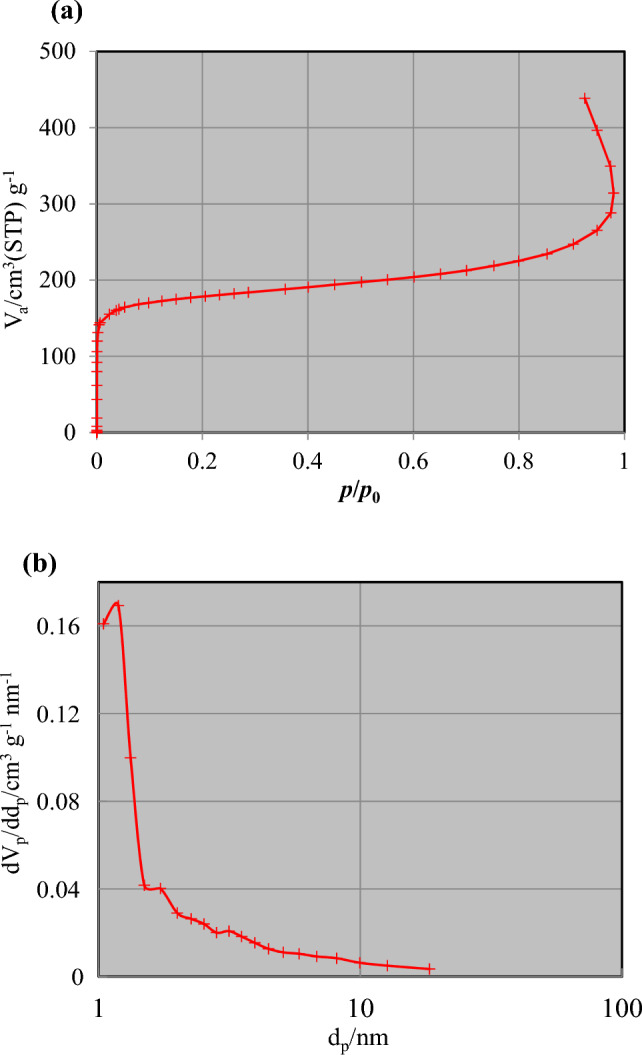
(**a**) Adsorption/desorption isotherm, and (**b**) BJH-plot adsorption branch, adsorptive N2, adsorption temperature 77 K of the prepared PET-MIL-53 (Al).

Figure 6 displays the nitrogen adsorption–desorption isotherms, pore diameter, and pore volume of the prepared PET-MIL-53 (Al). The pore size distribution reveals that the prepared catalyst exhibits pores with a diameter 4.2 nm. A pore diameter of 4.2 nm suggests that the catalyst possesses a well-defined pore structure in the mesoporous range. Mesopores typically have diameters ranging from 2 to 50 nm, and they offer advantages such as improved diffusion of molecules and accessibility to active sites within the catalyst. The presence of mesopores with a diameter of 4.2 nm suggests that the catalyst may have enhanced surface area and improved mass transfer properties compared to materials with smaller or larger pore sizes. These mesopores can provide pathways for reactant molecules to access the active sites of the catalyst, allowing for more efficient chemical reactions. The BET-specific surface area of the prepared Al-MOF catalyst is measured to be 666 m^2^/g, while the total pore volume is determined to be 0.6782 cm^3^/g. These values highlight the highly porous nature of the material. It is worth noting that the presence of mesopores with a larger surface area would expose more active sites, which could potentially enhance the catalytic activity of the prepared material.

### Catalytic performance of PET-MIL-53 (Al) in ODS process

The catalytic performance of as-synthesized PET-MIL-53(Al) was evaluated in the oxidative removal of thiophene using hydrogen peroxide as an oxidant. The different operating parameters were studied and were optimized.

### Effect of reaction time on % sulfur removal

**Figure 7:**
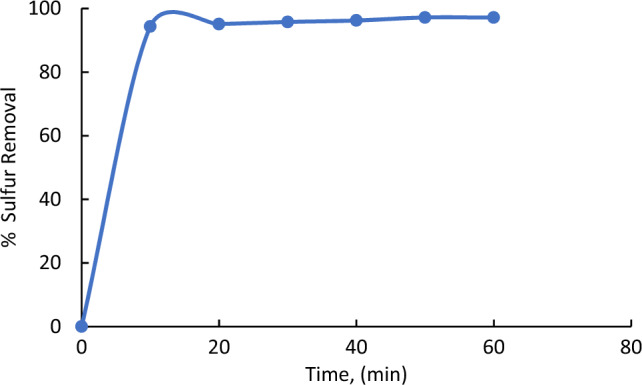
Effect of reaction time on % sulfur reduction. The experiments conditions: catalyst dose = 0. 5 g; model oil, 50 mL; acetonitrile, 50 ml; 25 °C. Oxidant to oil ratio (O/L):2:1.

Figure 7 illustrates the effect of reaction time on the % sulfur reduction for the investigated crude oil. The tests were carried out at six levels of reaction times, 10, 20, 30, 40, 50, 60 min. while the other operating parameters were kept constant as following, oxidant (H_2_O_2_) to oil ratio, 2:1, at ambient temp and catalyst dosage was 0.5 g. As illustrated in Fig. 7, the sulfur removal increases with increasing the reaction time from 10 to 40 min and finally approaches a constant value. Increasing reaction time increases the cavitation, producing fine emulsions, more converting of sulfur-containing compounds, and increasing the sulfur removal^24^. As the increase in the % sulfur removal was nearly the same for 30 and 40 min we choose 30 min to be the optimum reaction time.

#### Effect of oxidant to oil ratio (O/L)

**Figure 8:**
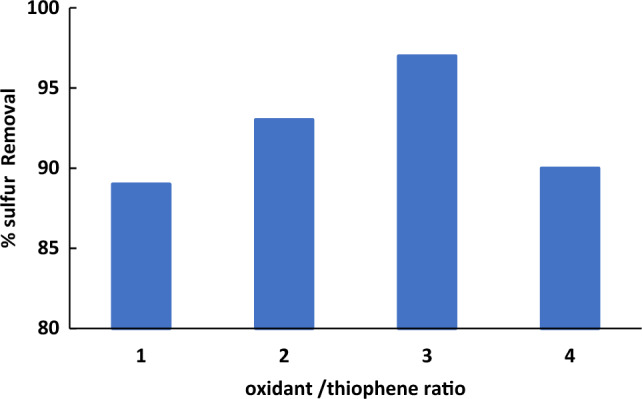
Effect of oxidant to oil ratio on oxidative desulfurization of thiophene. The experiments conditions: catalyst, 0. 5 g; model oil, 50 mL; acetonitrile, 50 ml; t, 30 min; 25 °C.

To investigate the effect of the oxidant dosage, the experiments were carried out under various H_2_O_2_/thiophene (O/L) molar ratios. As given in Fig. 8, sulfur reduction increased by increasing H_2_O_2_ amount. That can be returned to the increase in the production of the catalytic active species quantity. However, using higher amount of H_2_O_2_, decreases the % sulfur removal. This may confirm the fact that water has a destructive effect on the desulfurization as the amount of the introduced water increased with increasing hydrogen peroxide dosage. Also, excess H_2_O_2_ improve the hydrogen bond between the catalyst particles in the acetonitrile phase, leading to the transfer of the agglomerated catalyst into the oil phase, and thus its less availability for ODS which accordingly resulted in lower desulfurization rate^19,25^.

#### Effect of catalyst dose on sulfur oxidation

**Figure 9:**
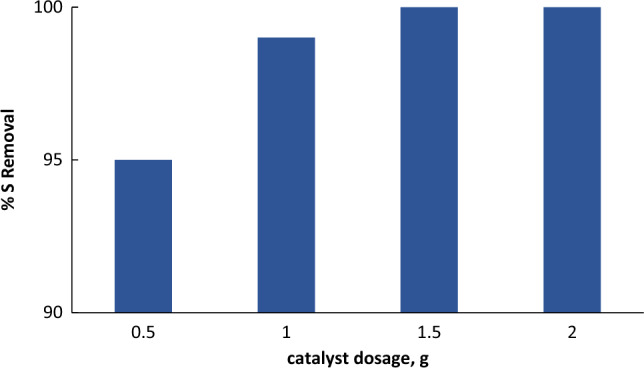
*E*ffect of catalyst dosage on oxidative desulfurization, the experiments conditions: time = 30 min; model oil, 50 mL; acetonitrile, 50 ml; O/L = 3:1; 25 °C.

Figure 9 illustrates the catalyst dose effect on % sulfur removal of the model oil. The experiments were investigated using four doses of catalyst, typically 0.5, 1, 1.5 and 2 g. The other parameters were kept constant. The effect of catalyst dosage on the thiophene oxidation was considered. As shown in the figure, thiophene reduction was improved with increasing catalyst amount. This can be credited to the increase in catalytic active sites^18,26^. Seemingly, 1.5 g of catalyst had offered enough catalytic active sites to achieve complete removal of thiophene.

#### Effect of ultrasounds

**Figure 10:**
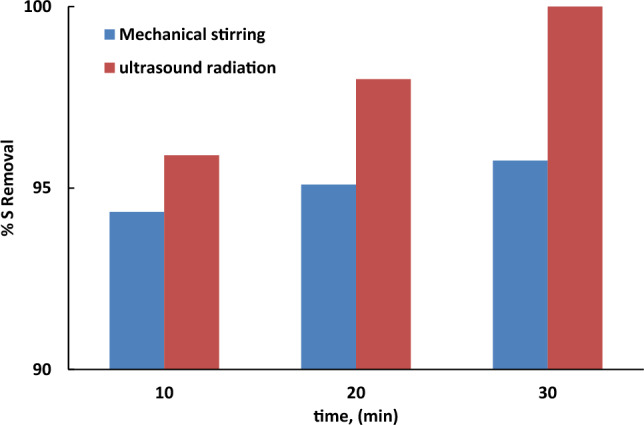
The effect of sonication on the sulfur removal. The experiments’ conditions: catalyst dosage = 0. 5 g; model oil, 50 mL; acetonitrile, 50 ml; (O/L) = 3:1; 25 °C.

Usually, there is a difficulty to completely mix the aqueous phase and the oil phase using normal mechanical stirring. Thus, the effect of ultrasounds was studied by exposing the reaction medium to ultrasound radiation and its effect was compared with that obtained from mechanical mixing. Results showed in Fig. 10 indicate that the % removal of sulfur was 100% on using ultrasound while it was 95.7% using mechanical stirring under the studied conditions. This may be attributed to the physical influence of the ultrasounic waves which provide mechanical action, cavitations and generate heat that can form a microemulsion between the water phase and the oil phase, thereby enhancing the mutual interaction between the molecules. This led to accelerate the oxidation process^25,26^.

## Case study: oxidative desulfurization of real crude oil

**Figure 11:**
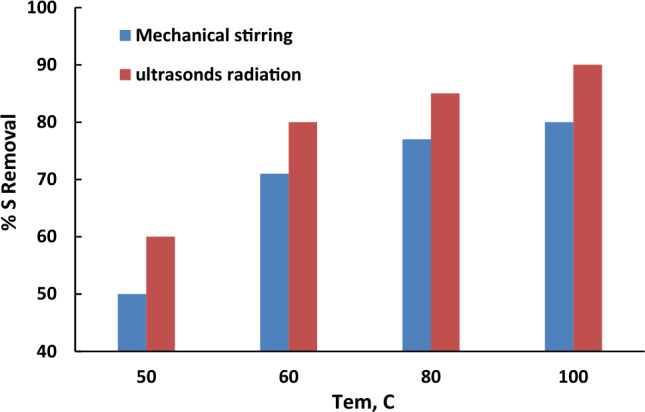
Effect of preheating temperture on the % sulfur removal. The experiments’ optimum conditions: catalyst dosage = 0. 5 g; model oil, 50 mL; acetonitrile, 50 ml; (O/L) = 3:1.

The previous determined optimum conditions for oxidative desulfurization of the studied model oil (thiopene in n-hexane) were applied to remove sulfur from a real crude oil supplied by Alexandria Petroleum Company. As the real crude has very high viscosity it was a must to heat the crude before the oxidation process. As shown in Fig. 11 three different temperatures were studied (60, 80 and 100 °C) while the other optimized parameters were remained constant. According to the results given in the figure, the sulfur removal increased with raising the preheating temperature from 50 to 100 °C. This may be returned to that increasing temperature reduce the liquid surface tension and thus increasing the creation of cavitations, resulting in increasing the oxidative desulfurization rate^29,30^. Figure 10 shows that the highest removal achieved was 90% at a preheating temperature of 100 °C in presence of ultrasound waves.

## Recycling of spent catalyst

**Figure 12:**
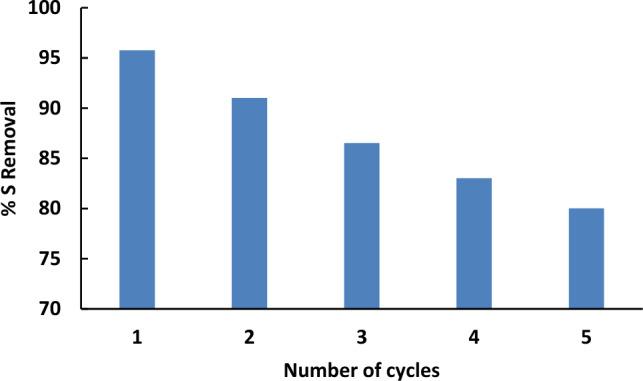
Recycling of the used catalyst on the sulfur removal in the oxidative desulfurization.

Spent catalyst has been separated after each experiment by centrifugation and has been washed with acetonitrile to remove the adsorbed oxidation product (sulfone), then it was dried for 24 h under vacuum for regeneration. The regenerated catalyst was reused for removing thiophene using the pre-determined optimum operating parameter for the oxidative desulfurization process. As is shown in Fig. 12, the catalytic performance of the catalyst decreased to 80% after five cycles.

It worth mentioned that, repeated regeneration of a catalyst can lead to a decrease in catalyst activity due to several factors:

Loss of active sites: each regeneration cycle can result in the loss or deactivation of active sites on the catalyst surface. Accumulation of catalyst fouling or contamination: despite regeneration efforts, contaminants or fouling agents can accumulate on the catalyst surface over multiple regeneration cycles. Residual impurities, reaction byproducts, or deposits from previous reactions may not be completely removed, leading to the blocking of active sites or hindrance of reactant diffusion. This accumulation can gradually reduce the overall catalytic activity of the catalyst. Catalyst degradation or structural changes: repeated regeneration cycles can cause structural changes or degradation of the catalyst material itself.

## Proposed mechanism of oxidative desulfurization using PET-MIL-53 (Al)

**Figure 13:**
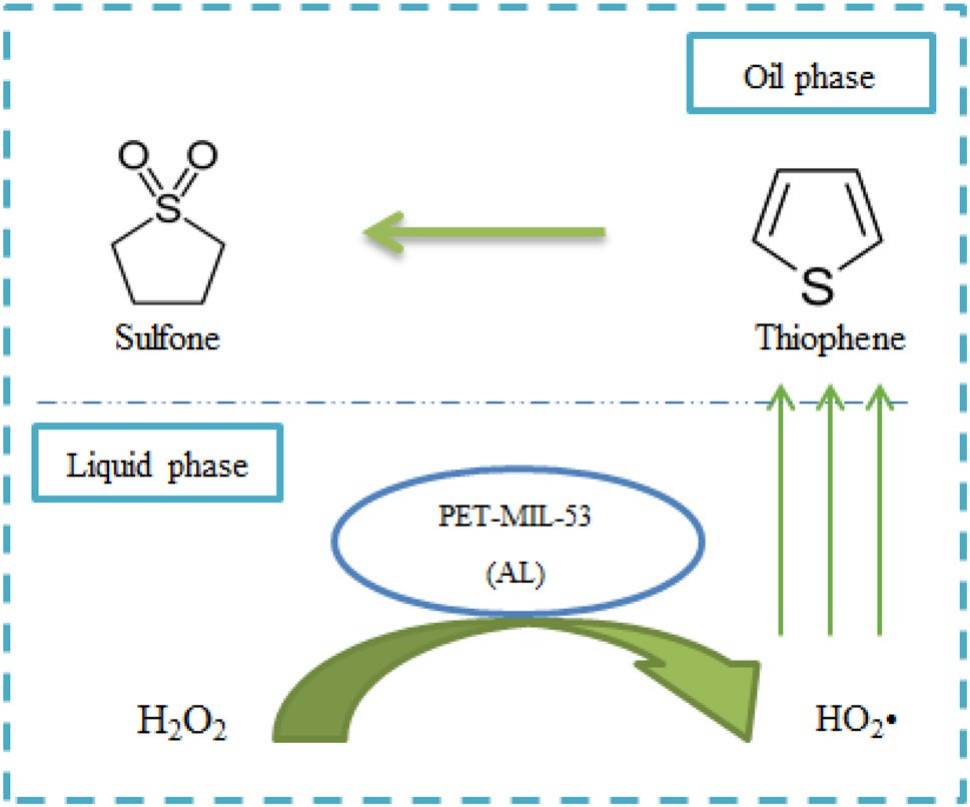
Proposed mechanism of oxidative desulfurization of thiophene using PET-MIL-53 (Al).

Figure 13 illustrates oxidation of thiophene during the oxidative desulfurization process, first, active free radicals (HO_2_^•^) were generated by the reaction of H_2_O_2_ with the Brønsted acidic sites in in the prepared PET-MIL-53 (Al) as a catalyst. Then, thiophene was oxidized to the corresponding sulfones by the formed radicals. Due to their high polarity, the formed sulfones could be easily removed from the model oil phase by acetonitrile as a polar solvent.

**Figure 14:**
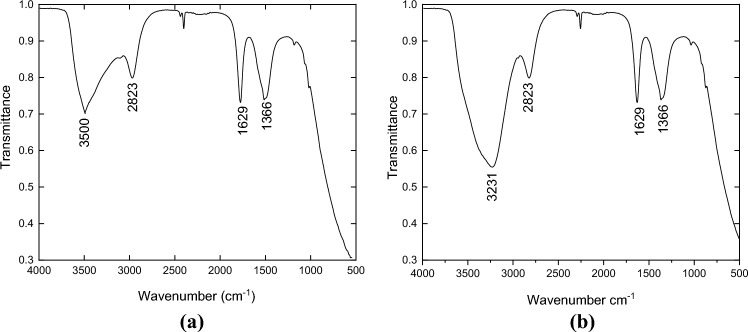
ATR-FTIR spectra of the reaction mixture (**a**) before adding H_2_O_2_, (**b**) 5 min after H_2_O_2_ addition. Reaction conditions: (Acetonitrile 40 ml, hydrogen peroxide 120 ml, oil 40 ml, catalyst dosage 0.5 g).

To ensure that OH radicals are the responisible for the catalytic reaction, ATR-FTIR test was performed for the reaction mixture before and after addition of H_2_O_2_ and the start of the reaction (after 5 min). From the obtained FRIR spectrum given in Fig. 14, it was obvious that there is an increase in the intensities of OH stretching, which can be explained by the formation of OH radicals after the addition of H_2_O_2_.

## Conclusions

The application of green synthesized Al-based MOF using waste PET bottles as a source for the organic linker in the oxidative desulfurization of crude oil results in a significant reduction in the quantities of the organic sulfur compounds. The effects of reaction temperature, reaction time, and amount of oxidant to oil molar ratio on the desulfurization rate were studied and optimized. Also, the effect of ultrasonic waves is compared with that under mechanical mixing. The results showed that ultrasounds exhibit a positive effect, where the catalyst dosage was about 0.5 g and H_2_O_2_/Oil ratio was 1:3 at 50 °C for 30 min.

## Data Availability

The datasets used and/or analysed during the current study available from the corresponding author on reasonable request.
